# Endocrine-Disrupting Activities of Flavones on Steroid Receptors: Structural Requirements and Synthesis of Novel Flavone with Improved Estrogenic Activity

**DOI:** 10.3390/biomedicines13030748

**Published:** 2025-03-19

**Authors:** Steven K. Nordeen, Vijay Kumar, Betty J. Bona, Joshua D. Batson, Donald S. Backos, Michael F. Wempe

**Affiliations:** 1 Department of Pathology, University of Colorado Anschutz Medical Center, Aurora, CO 80045, USA; 2 Department of Pharmaceutical Sciences, University of Colorado Anschutz Medical Center, Aurora, CO 80045, USA; vijay82cdri@gmail.com (V.K.); joshua.batson@cuanschutz.edu (J.D.B.); donaldbackos@gmail.com (D.S.B.); michael.wempe@kysu.edu (M.F.W.); 3 University of Colorado Cancer Center, University of Colorado—Anschutz Medical Campus, Aurora, CO 80045, USA

**Keywords:** estrogen receptor, androgen receptor, progesterone receptor, glucocorticoid receptor, flavonoid, flavone, endocrine disruptor

## Abstract

**Background/Objectives:** Flavonoids are common ubiquitous components of plants and are consumed by humans and livestock in their diets. Many different activities have been proposed for a variety of flavonoids that play a role in the benefits of a plant-rich diet. On the downside, excessive exposure to some flavonoids comes with a risk of endocrine disruption. Our objective was to define the structural elements of flavones and selected other flavonoids required for endocrine-disrupting activities on each of four steroid receptors, estrogen, androgen, progesterone, and glucocorticoid receptors. **Methods:** This work presents a systematic screen for the hormone agonist or antagonist activity of a selected panel of flavonoids on estrogen, androgen, progesterone, and glucocorticoid receptors. The screen is focused on the positional requirements of hydroxyl substituents on the flavone backbone. **Results:** Each receptor exhibited a distinct pattern for structural requirements of the flavones to impact receptor signaling. The most active flavones exhibited antagonist activity on androgen and progesterone receptors with an IC_50_ of 0.5 and 2 µM, respectively. Flavones only exhibited weak antagonism on glucocorticoid receptors. When active, flavones acted as estrogen receptor agonists. The findings were utilized to design and synthesize a novel flavone, 3-fluoro, 6,4′-dihydroxyflavone **14**, that displays increased potency as an estrogen agonist (EC_50_~30 nM). Modeling of the binding of this novel flavone predicts increased preference for ERα versus ERβ relative to the estrogenic phytoestrogen, genistein. **Conclusions:** The structural requirements for flavones to act as estrogen agonists and antagonists of other steroid receptors are defined. The synthesis of a novel flavone offers potential for topical applications where systemic estrogen activity is undesired. However, the results highlight the potential for endocrine disruption when certain flavones are consumed in quantity as supplements.

## 1. Introduction

Flavonoids are one of four major classes of polyphenolic compounds produced abundantly in plants. The function of flavonoids in plants, like many other polyphenolic compounds, is often not well understood, but given the well-established benefit of plant-rich diets in humans [1,2,3,4,5,6], their role as micronutrients in the human diet has been the focus of much attention. Although different flavonoids have been proposed to possess a wide variety of beneficial properties, notably anti-inflammatory, antioxidant, and anti-proliferative activities, it has been difficult to definitively establish a role in vivo, despite evidence supporting myriad activities in cell culture assays.

Multiple factors contribute to the difficulty in verifying the part that individual flavonoids may play in the benefits of a plant-rich diet. Plants make a bewildering array of polyphenolic compounds, and this composition varies between species and, for a given species, can depend on growth conditions. The use of plants in Asian medicine is non-standardized, making it difficult to pinpoint the contribution of a given plant, much less a single component. Furthermore, the bioavailability of flavonoids is generally poor due to metabolism by intestinal biota, poor intestinal uptake, and rapid modification by liver enzymes [7,8,9,10,11]. Therefore, circulating levels rarely reach the levels of flavonoids used in cell culture assessments. Nonetheless, the advertising and sale of flavonoid-containing nutraceuticals is robust despite the lack of clinical evidence for their benefit (or detriment).

Flavonoids can act as endocrine disruptors [12]. Many flavonoids are phytoestrogens, binding to estrogen receptors and activating their transcription-regulating activity. The consequences of excessive consumption of flavonoids were recognized more than 75 years ago in reports that observed reproductive abnormalities that mimicked effects of estrogens in sheep and cattle who had fed on fields of clover (*Trifolium subterraneum*) [13,14,15]. Isoflavones, notably genistein, were identified as active agents, and genistein was subsequently shown to bind to estrogen receptors and to stimulate the growth of estrogen-dependent tumor cells [16,17,18].

The interaction of flavonoids with steroid receptors other than the estrogen receptor has not received the same degree of attention. Nonetheless, different flavonoids have been reported to act as antagonists or agonists of glucocorticoid [19,20], androgen [19,20,21], and progesterone receptors [12,20,22,23,24,25]. In many cases, the EC_50_ is greater than 10 μM, calling into question whether such activities are relevant in any but exceptional circumstances outside a laboratory setting.

Here, we present the results of a screen where the activity of selected flavonoids has been examined on transcriptional activation by estrogen (ER), androgen (AR), progesterone (PR), and glucocorticoid (GR) receptors. Many flavonoids are weak agonists of the estrogen receptor, although the most active support significant induction in the tens of nanomolar range. On the other receptors, flavonoids often behave as antagonists. The most potent exhibit an IC_50_ at concentrations in the hundreds of nanomolar range.

Using the information gained from the structure–activity studies of flavones as estrogen receptor agonists, we designed and synthesized a pair of novel flavones. One of these, 3-fluoro, 6,4′-dihydroxyflavone, displayed superior potency for activating estrogen receptor-dependent transcription. We discuss the potential applications of this novel compound.

## 2. Materials and Methods

### 2.1. Chemicals

With the exception of the two novel flavones whose synthesis is described below, all flavonoids were procured from commercial vendors. Luteolin was obtained from R&D Systems Inc. (Minneapolis, MN, USA). All other flavones were purchased from Indofine Chemical Company Inc. (Hillsborough, NJ, USA). The tested compounds were solubilized in DMSO at 20 mM. Anhydrous acetone, anhydrous benzene, boron tribromide (BBr_3_), boron trifluoride (BF_3_) dibutyl etherate, deuterated dimethyl sulfoxide (DMSO-d_6_), deuterated chloroform (CDCl_3_), 1-(2,5-dihydroxyphenyl)ethenone, dimethyl sulfate (CH_3_)_2_SO_4_, absolute ethanol (EtOH), 3-fluoro-4-methoxy-benzoic acid, 4-hydroxy-benzaldehyde, 1-(2-hydroxy-5-methoxyphenyl)ethanone, hydroquinone, iodomethane, p-methoxybenzoyl chloride, oxalyl chloride, propionyl chloride, anhydrous pyridine, Selectfluor, sodium hydride (60% dispersion in mineral oil), sodium thiosulphate (Na_2_S_2_O_3_), trichlorofluoromethane (CFCl_3_), and triethylamine (TEA) were procured from Sigma-Aldrich Chemical Company (St. Louis, MO, USA). Acetic acid (AA), acetic anhydride, anhydrous acetonitrile (ACN), chloroform (CHCl_3_), dichloromethane (DCM), diethyl ether, anhydrous dimethylformamide (DMF), dimethylsulfoxide (DMSO), ethyl acetate (EtOAc), hexanes (Hex), hydrochloric acid (HCl), anhydrous magnesium sulfate (MgSO_4_), methanol (MeOH), potassium carbonate (K_2_CO_3_), potassium hydroxide (KOH), sodium bicarbonate (NaHCO_3_), sodium carbonate (Na_2_CO_3_), sodium chloride (NaCl), sodium hydroxide (NaOH), anhydrous sodium sulfate (Na_2_SO_4_), sulfuric acid (H_2_SO_4_), and HPLC grade water (H_2_O) were purchased from Fisher Scientific (Pittsburgh, PA, USA). Reactions were monitored via silica gel IB2-F thin-layer chromatography (TLC) plates from J.T. Baker (Phillipsburg, NJ, USA).

### 2.2. Cell Lines

The engineered T47D breast cancer cell lines employed for the assessment of flavonoid activity on glucocorticoid receptor (GR) and progesterone receptor (PR) signaling permitted the facile quantitation transcription of both an endogenous gene and a stably integrated reporter gene. The non-specific alkaline phosphatase gene is robustly induced by both glucocorticoids and progestins [12,26]. The T47D (A1-2) line has a stably integrated luciferase reporter gene controlled by a mouse mammary tumor virus promoter that is robustly induced by glucocorticoids [27]. The T47D K5pLuc cell line responds to progestins via induction of a stably integrated cytokeratin 5-luciferase reporter gene [28]. The T47D KBluc cell line has a stably integrated estrogen-response promoter driving firefly luciferase [29] and was used to assess the effects of the test compounds on estrogen receptor (ER)-mediated transcription. For assessment of the effect of the test compounds on androgen receptor (AR) signaling, we used the HeLa13 cell line engineered to stably express human AR and a PSA-luciferase reporter gene containing a 4× multimerized PSA upstream enhancer ARE1 linked to the E4 TATA box and luciferase gene [30].

### 2.3. Cell Culture

For assessment of the effect of flavones on glucocorticoid receptors, T47D (A1-2) cells were maintained in Minimal Essential Medium, 10% Fetal Bovine Serum (FBS), 0.2 mg/mL G418, penicillin (100 U/mL), and streptomycin (100 μg/mL). For experiments, medium was removed, and cultures rinsed with Phosphate-Buffered Saline (PBS) + 1.0 mM EDTA (PBSE), detached with trypsin, pelleted, and resuspended in medium to a concentration of 500,000 cells/mL. Aliquots of cell suspension, 100 microliters/well, were transferred into a 96-well, white, flat-bottomed plate and allowed to attach for 24 h. The following day, each well was treated with a 50 microliter aliquot of a test compound diluted in medium. Controls received medium containing DMSO diluted in the same way. For hormone treatment, dexamethasone was diluted in medium and added as 50 microliter aliquots so that the final volume in each well was 200 microliters. The final concentration of dexamethasone was 1.0 nM. Controls received medium without hormone. Hormone treatment was for 20 h at which time cells were harvested for assay of alkaline phosphatase and luciferase.

Experiments testing the effect of flavonoids on progesterone receptor-mediated transcription employed T47D K5pluc cells. The protocol was the same as that for T47D (A1-2) cells except the medium was Dulbecco’s Modified Eagle’s Medium supplemented with 10% FBS, insulin (10 nM), puromycin 200 ng/mL, nonessential amino acids, penicillin (100 U/mL), and streptomycin (100 μg/mL). When treated with hormone, the synthetic progestin, R5020, was used at 1.0 nM. Hormone treatment was for 20 h at which time cells were harvested for assay of alkaline phosphatase and luciferase.

Experiments testing the effect of flavonoids on estrogen receptor-mediated transcription employed T47D KBluc cells. These cells were maintained in RPMI medium with 10% FBS, penicillin (100 U/mL), and streptomycin (100 μg/mL). For reporter gene experiments, cells were detached as above and replated at 50% confluency in phenol red-free RPMI media with 10% charcoal-stripped FBS and antibiotics (day one). All subsequent steps used phenol red-free medium and trypsin. On day three, the medium was replaced with fresh medium. On day five, cells were washed with PBSE, detached, resuspended, diluted, and replated in the phenol red-free RPMI with charcoal-stripped serum (50,000 cells per well as above). Hormone, 17β estradiol (1.0 nM), and test compounds were added the following day and cells were harvested 20 h after treatment for assay of luciferase.

Experiments testing the effect of flavonoid on androgen receptor-mediated transcription employed HeLa 13 cells maintained in MEM medium supplemented with 10% FBS, 0.5 mg/mL G418, 0.05 mg/mL Hygromycin B, (100 U/mL), and streptomycin (100 μg/mL). For reporter gene experiments, cells were replated at a 1:5 dilution in MEM medium with 5% charcoal-stripped FBS and the antibiotics mentioned above. The medium was changed the next day. After 48 h, the cells were washed with PBSE, detached with phenol red-free trypsin, pelleted, resuspended and plated in 96 well plates as above. Hormone and flavonoid treatments were performed as described above with the HeLa 13 medium and charcoal-stripped FBS. When treated with hormone, the synthetic androgen, R1881, was used at 1.0 nM. Hormone treatment was for 20 h at which time cells were harvested for assay of luciferase.

### 2.4. Quantification of Reporter Gene Activity

For all cell lines, cell harvesting and the luminescence assay for alkaline phosphatase and/or luciferase were performed as previously described in detail [12]. Each condition was performed in quadruplicate. Percent induction is defined as the ratio of net activity observed in cells treated with hormone and flavonoid over the net activity measured in cells treated with hormone alone multiplied by 100. Percent inhibition is 100 minus the percent induction. Net activity is the activity in hormone-treated cells minus that of the same condition without hormone.

### 2.5. Computational Modeling

All molecular modeling calculations were conducted using Flare 9.0 (Cresset Biosciences, Ltd., Cambridgeshire, SG8 0SS, UK). X-ray crystal structures of human ERα (PDB ID: 1X7R) and ERβ (PDB ID: 1X7J) [31] were downloaded from the Protein Data Bank. Proteins were prepared and molecules were docked to the genistein binding site using the extra precision docking algorithm, and the highest scoring poses were selected for further analysis. MM/GBSA point energies were calculated for each ligand binding mode to predict the binding free energies for each compound.

### 2.6. Chemistry

**Scheme 1:**
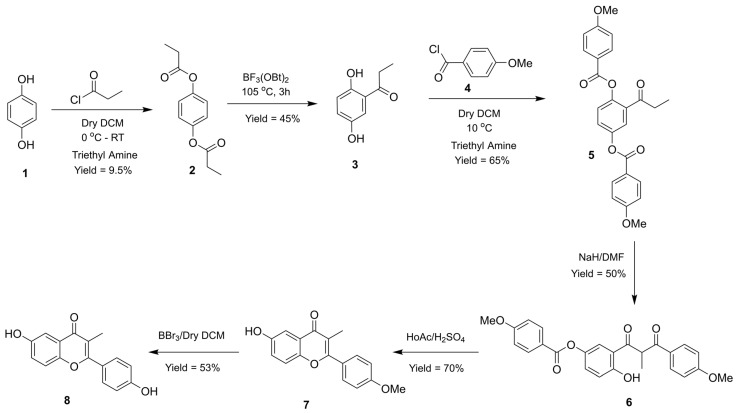
Synthesis of 3-methyl 6,4′-dihydroxyflavone (**8**).

**Scheme 2:**
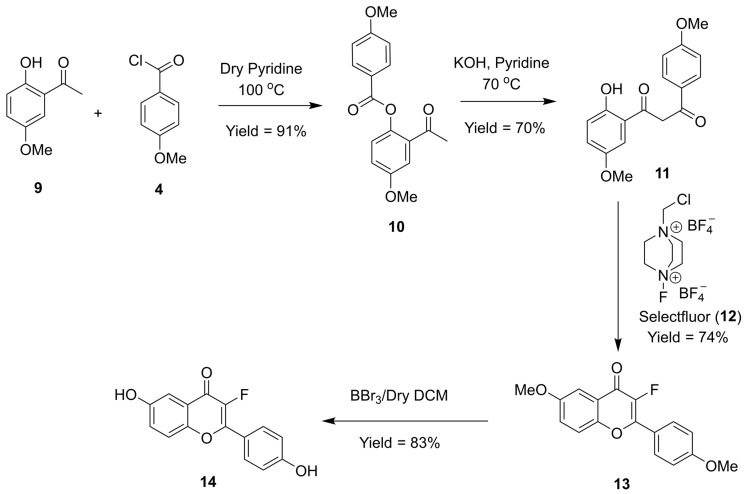
Synthesis of 3-fluoro 6,4′-dihydroxyflavone (**14**).

The synthesis for the novel flavone 3-methyl 6,4′-dihydroxyflavone (**8**) is depicted in Scheme 1. Scheme 2 depicts the synthesis strategy for the novel flavone 3-fluoro 6,4′-dihydroxy (**14**). Detailed methods and validation for the entire synthesis of both novel flavones are presented in the Supplementary Materials.

## 3. Results

**Figure 1:**
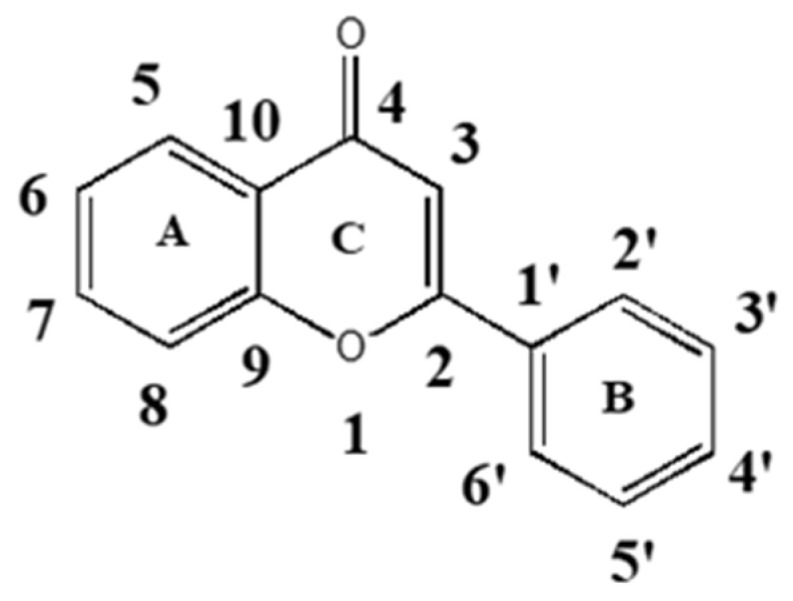
Flavone structure.

We previously reported that the flavone luteolin could act as a multifunctional endocrine disruptor [12]. Here, we present a systematic structure–activity study that investigates the activity of both natural and synthetic flavonoids (focusing on flavones) to act as agonists or antagonists on four different steroid receptors, estrogen receptors (ERs), androgen receptors (ARs), progesterone receptors (PRs), and glucocorticoid receptors (GRs). The structure of flavone and the standard numbering system of positions on the ring structures are depicted in Figure 1. The steroid receptors are a subgroup of the larger nuclear receptor family. The four steroid receptors, which include ARs, GRs, PRs, and the mineralocorticoid receptor, form a subset divergent from estrogen receptor (ER) alpha and ER beta. The ARs/GRs/PRs/MRs all have the capacity to bind to a similar DNA sequence. Each member of this subgroup also shares the ability to bind certain ligands that bind to one or more of the other members of this subfamily.

### 3.1. Flavonoid Antagonism of PR- and GR-Mediated Induction of Transcription

Based on our previous experience with PRs [12], the screen employed high doses (4 and 8 micromolar) to identify even weak activity. As detailed above, induction of both the endogenous alkaline phosphatase gene and a stably integrated luciferase genedriven by a hormone-responsive promoter was assessed. In most, but not all cases, both the endogenous alkaline phosphatase and luciferase reporter genes exhibited a similar response to a given compound. With the exception of apigenin (discussed below), no compound by itself exhibited significant agonist activity for either reporter gene. Many of the tested compounds had weak antagonist activity against PRs and/or GRs in the presence of a ligand (R5020 for PRs, dexamethasone for GRs). Table 1 presents the results of screening a series of flavonoids for their ability to inhibit ligand-induced transcription of the two reporter genes.

**Figure 2:**
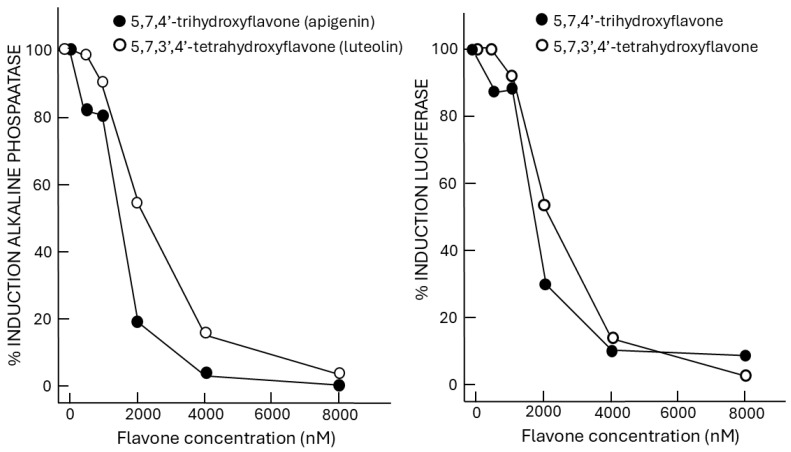
Inhibition of PR signaling by flavones.

Three compounds, chrysin (5,7-dihydroxyflavone), luteolin (5,7,3′4′-tetrahydroxyflavone), and apigenin (5,7,4′-trihydroxyflavone), inhibited PR-mediated gene expression of both reporter genes by at least 70 and 90% at 4.0 and 8.0 micromolar, respectively (bold in Table 1). The latter two were examined in further detail. Figure 2 presents a representative dose–response study. Luteolin and apigenin inhibit PR signaling in response to the strong synthetic ligand, R5020, at both the endogenous alkaline phosphatase gene and the stable luciferase transgene driven by a mouse mammary tumor virus promoter. These two flavones, common in dietary consumption, inhibit PR-mediated induction of gene expression with an IC_50_ in the 1–2 micromolar range.

Curiously, apigenin was the only compound tested that had a significant effect on basal expression levels of either alkaline phosphatase or luciferase. This was observed at doses of 4.0 micromolar and above. At 8.0 micromolar, apigenin alone induced luciferase to 22% of the induction achieved with R5020. Apigenin also induced alkaline phosphatase but to a lesser extent (4% at 8.0 micromolar). In Figure 2, the contribution of the elevated basal activity due to apigenin has been subtracted to calculate the net hormone activity (filled circles). At 8.0 micromolar, the bulk of the measured reporter gene activity is contributed by the flavone alone. This could mean that, at this high dose, apigenin is acting as a PR agonist or, alternatively, it has activated some cellular signal transduction pathway that activates the cytokeratin 5 promoter and, to a lesser extent, the endogenous alkaline phosphatase promoter. Apigenin has previously been reported to have progesterone agonist activity at high doses [22,23] and, conversely, progestin antagonist activity in animal tumor studies [24,25]. This discrepancy may be explained by the present observations that apigenin exhibits antagonist action while, at higher doses, behaves as an apparent agonist.

In contrast to PR signaling, no flavonoid compounds exhibited significant inhibition of glucocorticoid-induced transcription of both reporter genes even at a high dose of 8.0 micromolar (Table 1), although there were instances of modest inhibition of one of the two reporters at high levels of some flavonoids.

For both PR- and GR-dependent gene expression, there were instances where the inhibition was negative. This occurs when the induction of the reporter is greater with compound + hormone than with hormone alone. This is generally only seen with one of the two reporters. The amplification of hormone-dependent gene expression by concurrent activation of different signaling pathways has previously been observed [32,33]. Activation of protein kinase A has been reported to convert RU486 from a glucocorticoid and progesterone antagonist to an agonist [34,35]. We speculate that certain flavonoids may be impacting cell signaling pathways that in turn stimulate superinduction by glucocorticoid and progesterone receptors.

### 3.2. Flavonoid Antagonism of AR-Mediated Induction of Transcription

**Figure 3:**
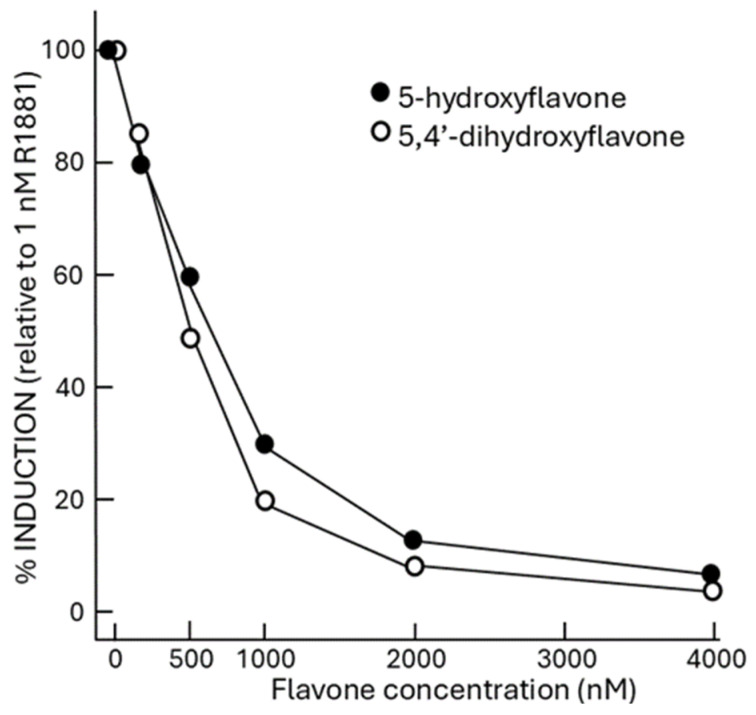
Inhibition of AR signaling by flavones.

For evaluation of the effect of flavonoids on ARs, the screen was conducted at concentrations of 1.0 and 4.0 micromolar, lower than those used for PRs and GRs. None of the tested compounds alone had a significant effect on the expression of the AR-dependent reporter gene but many could antagonize hormone (R1881)-dependent induction (Table 2). Using the same 70/90% inhibition of both reporter genes as a criterion, two flavones, 5-hydroxyflavone and 5,4′-hydroxyflavone, were identified as the most potent AR antagonists. Both exhibit an IC_50_ of about 0.5 micromolar (Figure 3). The latter is a multifunctional endocrine disruptor as it also has significant estrogen agonist activity (see Table 3).

### 3.3. Flavonoids as ER Agonists

The capacity of ERs to bind a wide variety of structurally distinct molecules, including different flavonoids, has been labeled as promiscuous but, as has been pointed out by Katzenellenbogen, is better described as “eclectic” [36]. High-affinity natural and synthetic ligands for ER alpha and beta ligands are distinct from ligands for the other steroid receptors, commonly possessing a pair of hydroxyl groups critical for high-affinity binding. As summarized in Table 3, many of the molecules tested exhibited at least some agonist activity at high concentrations and a number were quite active at sub-micromolar concentrations. The potency of a few flavonoids is comparable to isoflavones, like genistein, known to activate ERs. They are highlighted in bold font in Table 3.

The agonist activity of the most potent flavones was comparable to isoflavone phytoestrogens genistein, daidzein, and equol. Further comparisons of the activity of 6,4′-dihydroxyflavone and 3,6,4′-trihydroxyflavone at lower concentrations indicate that the latter exhibits an EC_50_ lower by 2–3 fold. This is consistent with our own modeling studies of flavone binding to estrogen receptors (see below) and a previous report that the affinity of 3,6,4′-trihydroxyflavone for ER is 3-fold greater than that of 6,4′-dihydroxyflavone and superior to a variety of other flavones [37]. The potency gained by a substitution at position 3 led us to investigate the role of substitutions at the 3 position further. To do so, we synthesized two analogs of 6,4′-dihydroxyflavone with either a methyl group (**8**) or a fluorine (**14**) at position 3. Both novel flavones were tested for estrogen agonist activity

**Figure 4:**
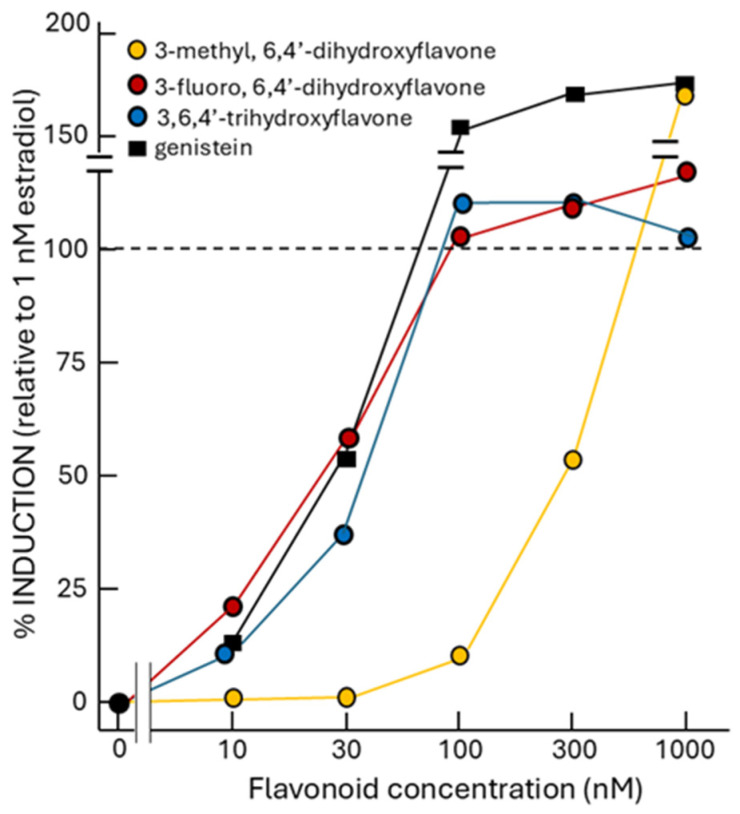
Induction of ER signaling by flavonoids.

As shown in Figure 4, 3-fluoro, 6,4′-dihydroxyflavone (**14**) exhibits robust estrogen agonist activity. Its potency is slightly but consistently better than that of 3,6,4′-trihydroxyflavone. The EC_50_ is comparable to that of the well-known phytoestrogen genistein. The potential for the use of 3-fluoro, 6,4′-dihydroxyflavone (**14**) in humans and its potential advantages over the phytoestrogen, genistein, are discussed below.

In contrast, the addition of a methyl group to 6,4′-dihydroxyflavone increases the EC_50_ by ten-fold or more. However, the 3-methyl, 6,4′-compound (**8**) now acts as a super-inducer at a high concentration (1.0 micromolar), stimulating reporter gene activity more than an optimal dose of estradiol. We often see a similar super-inducer phenomenon with isoflavones in this reporter system.

To better understand how different substitutions at the three position modulate estrogen receptor activity, the binding of 6,4′-dihydroxyflavones with H, OH, F, or CH_3_ at position 3 were modeled using the X-ray crystal structure of the isoflavone, genistein, bound to ER alpha and ER beta [24]. The predicted binding energies compared to those of genistein and the natural hormone, estradiol, are shown in Table 4.

The potency of a ligand is determined by a number of factors in addition to binding energy, including the particular output or activity measured, the cellular context including receptor density, the impact of post-translational modifications of the receptor, the co-factors present, and metabolism of the ligand. The 3-fluoro, 6,4′-dihydroxyflavone (**14**) has a predicted binding energy for ERα similar to that of 3,6,4′-dihydroxyflavone. Both are better binders than 6,4′-dihydroxyflavone and much poorer than the native hormone, estradiol, which is over 1000-fold more potent in the assays used here.

**Figure 5:**
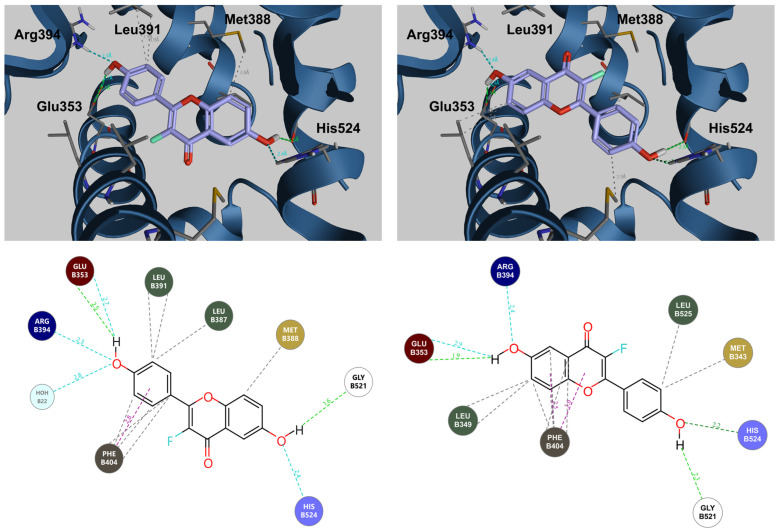
Three-dimensional and two-dimensional models of 3′-fluoro 6,4′-dihydroxyflavone (**14**) binding to ERα. Expected (**left**) and flipped (**right**) binding modes are depicted along with predicted contacts with ERα.

An unexpected finding of docking studies is that certain flavones are predicted to potentially bind to both forms of ERs in two orientations with similar energetics. The first is the orientation ‘expected’ from the crystal structure of genistein bound to ER alpha and ER beta. In the second, ‘flipped’, orientation, the flavone molecule is rotated 180 degrees so that the hydroxyl of the A-ring and the 4′ hydroxyl of B-ring effectively switch places (Figure 5). The potential contribution of this alternative binding mode is particularly notable for the 3-fluoro compound. The predicted binding energy of the 3-fluoro compound to ERα is superior to genistein, an isoflavone, in both the expected and flipped binding modes. Conversely with ERβ, genistein has a superior predicted binding energy compared to 3-fluoro, 6,4′-dihydroxyflavone (**14**).

Supplemental Figure S1 presents 3D and 2D interaction maps comparing the predicted positioning of 6,4′-dihydroxyflavone, 3,6,4′-trihydroxyflavone, 3,6,4′-trihydroxyflavone, and genistein (5,7,4′-trihydroxyisoflavone) and their predicted contacts in the ligand binding pocket of ERα. As might be expected, the positioning of 3-fluoro, 6,4′ dihydroxyflavone and 3,6,4′-trihydroxyflavone (**14**) are very similar in both the expected and the flipped orientations, and the contacts predicted, are virtually identical. The contribution made by a fluorine substitution at position 3 to binding energy is not readily apparent.

In order for the 4′OH group of the isoflavone and the novel fluoroflavone to make critical contacts with Arg 394 and Glu 353, the flavones with hydroxyl or fluorine substitutions at the 3 position are inverted about their x-axis relative to genistein to favorably reposition the A- and C-rings. While the contacts made by genistein and the flavones with F or OH at position 3 are virtually identical, there are consequences for the predicted binding energies of flavones to ERα versus ERβ, particularly the 3-fluoro compound, relative to genistein. The importance of these differences and the specificity for the alpha and beta forms of the estrogen receptor are discussed below.

## 4. Discussion

The ubiquity of flavones and other flavonoids in the human diet via the consumption of plants has justifiably prompted many investigations into the activities they possess. Many of the reported activities are consistent with their potential as contributors to the benefits of a plant-rich diet.

Direct demonstration of this conjecture has proven difficult due to the great variety of flavonoids in plants, their poor bioavailability, and rapid metabolism. The use of doses far beyond those reached, even transiently, in vivo means that many of the reported activities likely have little relevance in normal consumption. However, as first demonstrated by endocrine disruption in grazing animals [13,14,15], excessive consumption can result in metabolic effects, in this case deleterious endocrine-disrupting effects.

The nutraceutical industry represents a multibillion-dollar industry. Flavonoids are well represented among these supplements that are available over the counter. Given their availability, there is potential for consumption of flavonoids well beyond levels available through a normal diet. Thus, flavonoids represent a complex situation with potential for both benefit and harm.

In recognition of this, we screened a variety of flavonoids, focusing on flavones, to identify the features necessary for interaction with steroid receptor signaling pathways. Four steroid signaling pathways were examined using cell reporter assays. We took the extra step, when assessing glucocorticoid and progesterone signaling, of assessing a readily assayed endogenous gene, alkaline phosphatase, that is known to be induced by both glucocorticoids and progestins in T47D-derived cell lines [12,26]. Concurrently, a stably integrated reporter gene with a hormone-inducible promoter driving firefly luciferase was assayed.

Each of the receptor signaling pathways assessed has a distinct pattern of flavone interaction. For ERs, hydroxyl groups are highly preferred at positions 4′ and 6 or 7. For ARs, the hydroxyl at position 5 appears most critical to antagonist activity, whereas for PRs, hydroxyls at 5,7 and 4′ all contribute.

For ARs, PRs, and GRs, flavonoids tend to act as antagonists of hormone-dependent transcription. ARs are the most sensitive; the best flavones inhibit AR-mediated transcription with an IC_50_ of 0.5–1.0 micromolar. Selected flavones inhibit PRs with an IC_50_ between 1.0 and 2.0 micromolar, whereas with GRs, only modest inhibition, at best, was observed at both reporter genes even, at an 8.0 micromolar dose of a flavone.

In contrast, with ER signaling, active flavonoids behave as agonists, acting at low concentrations compared to ARs, PRs, and GRs. The most potent of the flavones has an EC_50_ of 30–100 nM, which was notably the novel synthetic flavone we report here, 3-fluoro, 6,4′-dihydroxyflavone (**14**). The EC_50_ of this compound is as low as the potent phytoestrogen, genistein, that can cause endocrine disruption when livestock graze on plants with high levels of this isoflavone.

It should be noted that T47D cells have been reported to express both estrogen receptor alpha and beta [38,39]. Therefore, the reporter assays assessed contributions from both forms of ERs. Genistein preferentially binds to ERβ over ERα [31,40]. It is notable that the predicted binding energy of the 3-fluoro compound to ERα is superior to that of genistein, especially in the flipped orientation, whereas the opposite is true for binding to ERβ. This suggests that 3-fluoro, 6,4′-dihydroxyflavone (**14**) has utility in circumstances where the activation of ERα activity is desired. For example, the epithelium of the menopausal vagina expresses little or no ERβ [41,42] but responds to estrogens via ERα, suggesting that local administration of 3-fluoro, 6,4′-dihydroxyflavone (**14**) would be more effective than genistein.

Further studies will explore the potential of the novel flavone we have synthesized, 3-fluoro, 6,4′-dihydroxyflavone (**14**), for human applications where systemic estrogen activity is undesirable. Such applications in women who are estrogen-deficient due to natural or therapy-induced menopause might include dermatological usage for skin restoration or for the reversal of vaginal atrophy that can result in symptoms of genitourinary syndrome of menopause such as vaginitis, increased vaginal tract infections, and dyspareunia (painful intercourse).

The strength of this work extends beyond the utility of defining the structural elements of flavones that permit interaction with four different steroid receptors. Flavones were identified that have the potential to mediate an endocrine-disrupting effect at physiologically relevant doses, especially when taken as supplements. In addition, information from the screen led to the synthesis of a novel flavone with increased estrogenic activity. The primary weakness of this work is that the activity data were all collected in cell culture. A report on experiments verifying the in vivo activity of the novel flavone reported here is in preparation.

## 5. Patent

The novel flavones reported here are the subject of patent application no. 18/833,240.

## Figures and Tables

**Table 1:** Effects of flavonoids on signaling by PR and GR.

Flavonoid	% Inhibition of PR Induction				% Inhibition of GR Induction			
	4 µM		8 µM		4 µM		8 µM	
	LUC	AP	LUC	AP	LUC	AP	LUC	AP
4′-hydroxyflavone	−62	31	−81	49	−43	49	−49	49
5-hydroxyflavone	−23	26	45	61	−9	38	−30	12
**5,7-dihydroxyflavone ^1^**	**71**	**78**	**90**	**89**	0	22	−6	38
5,4′-dihydroxyflavone	−12	23	80	74	13	34	13	33
6,4′-dihydroxyflavone	−147	39	−103	37	−44	26	−82	39
7,4′-dihydroxyflavone	−16	41	−49	55	−92	47	−38	64
3′,4′-dihydroxyflavone	−64	8	−14	27	−2	−15	−6	38
**5,7,4′-trihydroxyflavone ^2^**	**86**	**90**	**88**	98 *	6	−19	35	49 *
5,7,4′-trihydroxyflavanone ^3^	52	43	98	90	15	8	19	7
5,3′,4′-trihydroxyflavone	−7	46	55	65	−16	27	−24	40
7,3′,4′-trihydroxyflavone	−11	13	−10	39	−117	21	−87	36
**5,7,3′,4′-tetrahydroxyflavone ^4^**	**76**	**82**	**95**	**95**	−8	33	−3	55
5,7,3′,4′-tetrahydroxyflavanone ^5^	3	8	23	40	5	4	18	−4
5,7,3′-triOH, 4′methoxyflavanone ^6^	−6	8	16	28	nd	1	15	51
3,5,7,3′,4′-pentahydroxyflavone ^7^	47	52	78	79	35	−3	47	−19
5,7,3′4′,5′-pentahydroxyflavone	−97	12	−19	45	5	−19	−25	46
flavone	−66	10	−107	40	4	13	−24	34
5,7,4′-trihydroxyisoflavone ^8^	−96	49	−208	32	−7	22	nd	nd
7,4′-dihydroxyisoflavone ^9^	−13	41	−126	38	nd	nd	−52	5
7,4′-dihydroxyisoflavan ^10^	nd	nd	−88	42	nd	nd	nd	nd

Common names: ^1^ chrysin, ^2^ apigenin, ^3^ naringenin, ^4^ luteolin, ^5^ eriodictyol, ^6^ hesperetin, ^7^ quercetin, ^8^ genistein, ^9^ daidzein, and ^10^ equol. nd: not done. LUC: luciferase; AP: alkaline phosphatase. * Api-genin alone caused large induction of alkaline phosphatase activity that was subtracted from the calculation of inhibition.

**Table 2:** Antagonism of AR signaling by flavonoids.

% Inhibition of AR-Mediated Gene Induction		
Flavonoid	1 µM	4 µM
4′-hydroxyflavone	29	65
**5-hydroxyflavone**	**72**	**96**
5,7-dihydroxyflavone ^1^	−13	46
**5,4′-dihydroxyflavone**	**77**	**97**
6,4′-dihydroxyflavone	43	54
7,4′-dihydroxyflavone	−18	−20
3′,4′-dihydroxyflavone	40	75
5,7,4′-trihydroxyflavone ^2^	12	74
5,7,4′-trihydroxyflavanone ^3^	−17	9
5,3′,4′-trihydroxyflavone	29	91
7,3′,4′-trihydroxyflavone	−6	11
5,7,3′,4′-tetrahydroxyflavone ^4^	−20	67
5,7,3′,4′-tetrahydroxyflavanone ^5^	13	24
5,7,3′-triOH, 4′methoxyflavanone ^6^	−28	−30
3,5,7,3′,4′-pentahydroxyflavone ^7^	28	72
5,7,3′4′,5′-pentahdroxyflavone	−8	24
flavone	−12	55
4′-hydroxyflavanone	−8	0
6-hydroxyflavanone	−8	22
3,6-dihydroxyflavone	35	52
3,4′-dihydroxyflavone	3	27
5,4′-methoxyflavone	4	53
5-methoxy,4′-hydroxyflavone	−4	34
6-methoxy,4′-hydroxyflavone	27	49
6-hydroxy,4′-methoxyflavone	32	39
3,6,4′-trihydroxyflavone	53	71
3,7,4′-trihydroxyflavone	−6	39
3-hydroxy, 6,4′-dimethoxyflavone	−17	41
5,7,4′-trihydroxyisoflavone ^8^	55	22
7,4′-dihydroxyisoflavone ^9^	nd	24
7,4′-dihydroxyisoflavan ^10^	nd	22

Common names: ^1^ chrysin, ^2^ apigenin, ^3^ naringenin, ^4^ luteolin, ^5^ eriodictyol, ^6^ hesperetin, ^7^ quercetin, ^8^ genistein, ^9^ daidzein, and ^10^ equol. nd: not done. Compounds that inhibit AR signaling by 70 and 90% at 1 µM and 4 µM respectively are indicated by bold font.

**Table 3:** Percent induction of ER signaling relative to 1nM estradiol.

% ER Induction		
Flavonoid	1 µM	4 µM
4′-hydroxyflavone	12	130
5-hydroxyflavone	0	4
5,7-dihydroxyflavone ^1^	1	17
5,4′-dihydroxyflavone	53	138
**6,4′-dihydroxyflavone**	**119**	**192**
**7,4′-dihydroxyflavone**	**86**	**140**
3′,4′-dihydroxyflavone	0	13
5,7,4′-trihydroxyflavone ^2^	62	161
5,7,4′-trihydroxyflavanone ^3^	48	93
5,3′,4′-trihydroxyflavone	0	13
7,3′,4′-trihydroxyflavone	0	30
5,7,3′,4′-tetrahydroxyflavone ^4^	3	80
5,7,3′,4′-tetrahydroxyflavanone ^5^	0	20
5,7,3′-triOH, 4′methoxyflavanone ^6^	10	6
3,5,7,3′,4′-pentahydroxyflavone ^7^	3	12
5,7,3′4′,5′-pentahdroxyflavone	0	−1
flavone	1	3
4′-hydroxyflavanone	12	109
6-hydroxyflavanone	15	111
3,6-dihydroxyflavone	55	94
3,4′-dihydroxyflavone	13	125
5,4′-methoxyflavone	1	31
5-methoxy, 4′-hydroxyflavone	−2	10
**6-methoxy, 4′-hydroxyflavone**	**129**	**167**
6-hydroxy, 4′-methoxyflavone	63	131
**3,6,4′-trihydroxyflavone**	**102**	**108**
**3,7,4′-trihydroxyflavone**	**86**	**114**
3-hydroxy, 6,4′-dimethoxyflavone	2	3
**5,7,4′-trihydroxyisoflavone ^8^**	**125**	**180**
**7,4′-dihydroxyisoflavone ^9^**	**125**	nd
7,4′-dihydroxyisoflavan ^10^	96	84

Common names: ^1^ chrysin, ^2^ apigenin, ^3^ naringenin, ^4^ luteolin, ^5^ eriodictyol, ^6^ hesperetin, ^7^ quercetin, ^8^ genistein, ^9^ daidzein, and ^10^ equol. nd: not done.

**Table 4:** Predicted binding energies of estrogenic flavones and estradiol.

Estrogen Receptor Ligand	Binding Mode	Predicted Binding Energy (kcal/mol)	
		ERα	ERβ
6,4′-dihydroxyflavone	expected	−32.48	−27.24
	flipped	−30.97	−24.83
3,6,4′-trihydroxyflavone	expected	−37.85	−30.04
	flipped	−30.89	−23.51
3-methyl,6,4′-dihydroxyflavone	expected	−9.31	−17.12
	flipped	−18.16	−16.65
3-fluoro,6,4′-dihydroxyflavone	expected	−36.22	−29.45
	flipped	−33.81	−26.46
genistein (5,7,4′-trihydroxyisoflavone)	expected	−34.62	−30.29
	flipped	−27.36	−29.01
17β-estradiol	expected	−54.01	−41.35
	flipped	5.74	−1.27

## Data Availability

The raw data supporting the conclusions of this article will be made available by the authors on request.

## Supplementary Materials

The following supporting information can be downloaded at: https://www.mdpi.com/article/10.3390/biomedicines13030748/s1, Methods and validation for synthesis of 3-metthyl 6,4′dihydroxyflavone and 3-fluoro 6,4′dihydroxyflavone; Figure S1. Each pair depicts the ‘expected’ (left) and ‘flipped’ (right) binding mode of a flavonoid. Upper left, 3-fluoro 6,4′-dihydroxyflavone; upper right, genistein (5,7,4′-trihydroxyisoflavone) lower left, 3,6,4′-triihydroxyflavone, lower right, 6,4′-dihydroxyflavone.
