# Supplementary material for: Endocrine-Disrupting Activities of Flavones on Steroid Receptors: Structural Requirements and Synthesis of Novel Flavone with Improved Estrogenic Activity
Source: Biomedicines. 2025 Mar 19;13(3):748. doi: 10.3390/biomedicines13030748 (PMC11940309; doi:10.3390/biomedicines13030748)
Supplement: Supplementary file 1 [file biomedicines-13-00748-s001.zip › biomedicines-3395364-supplementary.pdf]

Supplementary Materials

Synthesis of 3-methyl, 6,4′-dihydroxyflavone.

1,4-phenylene dipropionate (2): Hydroquinone (**1**, 1.1 g, 10.0 mmol, 1.0 equiv.) and TEA (5.2 mL, 3.7 g, 37.0 mmol, 3.7 equiv.) were dissolved in 150 mL of dry DCM and cooled to 0°C. Propionyl chloride (3.23 mL, 3.42 g, 37.0 mmol, 3.7 equiv.) was then added dropwise to the solution while stirring. The reaction mixture was allowed to warm to room temperature (RT) while stirring overnight. The mixture was then washed three times with 1.0 M NaOH (20 mL) and once with brine (50 mL) and dried over Na_2SO_4. The material was filtered, and the solvent subsequently removed using rotary evaporation under reduced pressure, and the product was purified by silica gel chromatography using 10:1 hex/EtOAc to give 0.20 g of **(2)** as a white solid. ^1H NMR (400 MHz, $CDCl_3$): δ 1.26 (t, J = 7.6 Hz, 6H), 2.58 (q, J = 7.4 Hz, 2H), 7.09 (s, 4H) ppm.

1-(2,5-dihydroxyphenyl)propan-1-one (3): In a 100-mL round-bottom flask (RBF) equipped with a condenser and a stir bar were placed 4.0 mL (19.37 mmol) of BF_3 and (1.0 g, 4.50 mmol) of (**2**). Heat was then applied, and the mixture was refluxed at 105°C for 2.0h. The resulting solution was allowed to cool to RT, and then poured into cold H_2O (100 mL) and extracted with DCM (100 mL). The organic phase was washed again with H_2O (2 x 50 mL) and dried over $MgSO_4$. The material was filtered, and the solvent subsequently removed using rotary evaporation under reduced pressure, and the crude product was purified by silica gel chromatography using 30% acetone/hex to give 0.40 g (45%) of **(3)** as a white solid. mp 90-92°. ^1H NMR (400 MHz, $CDCl_3$): δ 12.03 (s, 1H), 7.23 (d, J = 2.7 Hz, 1H), 7.03 (dd, J = 8.9, 2.7 Hz, 1H), 6.87 (d, J = 8.9 Hz, 1H), 5.70 (s, 1H), 2.97 (q, J = 7.3 Hz, 2H), 1.21 (t, J = 7.3 Hz, 3H); ^{13}C NMR (100 MHz, $CDCl_3$) δ 207.06, 156.16, 147.69, 124.85, 119.25, 118.91, 114.84, 31.67, 8.16.

2-propionyl-1,4-phenylene bis(4-methoxybenzoate) (5): 2,5-Dihydroxypropiophenone (**3**, 1.7 g, 10.0 mmol, 1.0 equiv.) and TEA (5.2 mL, 3.7 g, 37.0 mmol, 3.7 equiv.) were dissolved in 150 mL of dry DCM and cooled to 0 °C. 4-Methoxybenzoyl chloride **(4)** (5.0 mL, 6.31 g, 37.0 mmol, 3.7 equiv.) was then added dropwise to the solution while stirring. The reaction mixture warmed to RT while stirring overnight. The mixture was then washed three times with 1.0 M NaOH (50 mL) and once with brine (50 mL) and dried over Na_2SO_4. The material was filtered, and the solvent was subsequently removed using rotary evaporation under reduced pressure and the product was purified by silica gel chromatography using 10:1 hex/EtOAc to give 2.8 g (65 %) of **(5)** as a white solid. ^1H NMR (400 MHz, $CDCl_3$): δ 8.17 – 8.09 (m, 4H), 7.66 – 7.59 (m, 1H), 7.42 – 7.37 (m, 1H), 7.25 – 7.22 (m, 1H), 7.00 – 6.95 (m, 4H), 2.92 – 2.84 (m, 2H), 1.11 – 1.06 (m, 3H) ppm.

4-hydroxy-3-(3-(4-methoxyphenyl)-2-methyl-3-oxopropanoyl)phenyl 4-methoxybenzoate (6): To a suspension of 84 mg (2.1 mmol) of sodium hydride in 10 mL of anhydrous DMF at 0-5 °C under N_2 was added a solution of 0.87 g (2.0 mmol) of (**5**) anhydrous DMF (2.0 mL) over 5 min. After 2.0 hr of stirring at 0-5°C, the reaction was quenched by the addition of acetic acid (0.2 mL). The reaction mixture was diluted with ethyl ether (20 mL) and saturated aqueous NaCl/H_2O (50 mL). The layers were separated, and the aqueous layer was extracted with ethyl ether (25 mL). The combined organic layers were washed with 1:1 saturated aqueous NaCl/H_2O (30 mL**),** and organic dried over anhydrous Na_2SO_4, filtered, and concentrated *in vacuo* to a solid residue. The solid was crystallized from refluxing EtOAc (100 mL) under nitrogen by slow addition of hexanes (200 mL) and subsequent cooling to RT and then to 0-5°C. The solid was collected by filtration and dried (2.0 h) under high vacuum (60 °C) to provide 0.43 g (50%) of **(6).** ^{1}H NMR (400 MHz, $CDCl_3$): δ 11.99 (s, 1H), 8.14 – 8.06 (m, 2H), 7.95 – 7.88 (m, 2H), 7.62 – 7.58 (m, 1H), 7.37 – 7.30 (m, 1H), 7.08 – 6.88 (m, 5H), 5.24 – 5.14 (m, 1H), 3.90 (s, 3H), 3.84 (s, 3H), 1.64 – 1.54 (m, 3H) ppm.

6-hydroxy-2-(4-methoxyphenyl)-3-methyl-4*H*-chromen-4-one (7): Compound **6** (0.43 g, 1.0 mmol) was suspended in acetic acid (3.0 mL) and H_2SO_4 (30 mL). After reflux (2.0 h) under N_2, the reaction was cooled to RT, diluted with Hex (10 mL), and filtered. The filter cake was washed with Hex and then dried overnight under vacuum. The solid was purified by flash chromatography (Hex/EtOAc, 1:1) to give 0.20 g (yield = 70%) of **(7)** as a white solid. ^{1}H NMR (400 MHz, DMSO *d*$_6$): δ 9.92 (s, 1H), 7.67 (d, *J* = 8.8 Hz, 2H), 7.51 (d, *J* = 8.8 Hz, 1H), 7.37 – 7.32 (m, 1H), 7.26 – 7.18 (m, 1H), 7.12 (d, *J* = 8.8 Hz, 2H), 3.86 (s, 3H), 2.04 (s, 3H) ppm.

6-hydroxy-2-(4-hydroxyphenyl)-3-methyl-4*H*-chromen-4-one (8): A Teflon-coated magnetic stir bar was added to an oven-dried, 100 mL RBF. Compound **(7)** (0.20 g, 0.71 mmol) in dry DCM (40 mL) was added and the mixture was cooled using an ice bath. After 20 min, BBr_3 (0.18 mL, 1.78 mmol, 2.5 equiv) was added at 0-5 °C and the reaction mixture was stirred at the same temperature (60 min). Then, the reaction mixture was warmed to RT and stirred (18 h). Reaction progress was monitored by TLC. After completion, saturated $NaHCO_3$ solution (50 mL) was added slowly, and the solution was extracted with DCM (50 mL x 2). The organic layers were combined and dried over anhydrous Na_2SO_4, filtered, evaporated, and chromatographed over silica gel eluting with Hex:EtOAc (1:4) to give **8** (100 mg, 53%). ^{1}H NMR (400 MHz, DMSO *d*$_6$): δ 10.04 (s, 1H), 9.86 (s, 1H), 7.56 (d, *J* = 8.8 Hz, 2H), 7.49 (d, *J* = 8.8 Hz, 1H), 7.37 – 7.32 (m, 1H), 7.24 – 7.18 (m, 1H), 6.94 (d, *J* = 8.8 Hz, 2H), 2.04 (s, 3H); ^{13}C NMR (100 MHz, DMSO *d*$_6$): δ 177.7, 161.2, 159.7, 154.9, 149.7, 131.1, 124.1, 123.3, 123.0, 119.9, 115.7, 114.9, 107.9, 12.2 ppm.

Synthesis of 3-fluoro, 6,4′-dihydroxyflavone **(14)** (*3-fluoro-6-hydroxy-2-(4-hydroxyphenyl)-4H-chromen-4-one*).

2-acetyl-4-methoxyphenyl 4-methoxybenzoate (10): A dry RBF equipped with a reflux condenser charged with **(9)** (3.32 g, 20.0 mmol), *p*-methoxybenzoyl chloride (**4**; 4.09 g, 24.0 mmol, 1.2 equiv), and dry pyridine (40.0 mL) was heated (100°C, 4.0 h). The mixture was then poured into a mixture of ice and 1.0 N HCl (70 mL) and extracted with EtOAc. The extract was washed with H_2O and aqueous Na_2CO_3 (50 mL) and dried over anhydrous Na_2SO_4. The material was filtered, and the solvent was evaporated under reduced pressure to give crude (**10,** 5.5g, 91% yield). Crude was used as such for next step.

1-(2-hydroxy-5-methoxyphenyl)-3-(4-methoxyphenyl)propane-1,3-dione (11); To a solution of the crude **10** (5.0 g, 16.66 mmol) in pyridine (30.0 mL) was added freshly powdered KOH (5.3 g), and the resultant mixture was vigorously stirred (70°C, overnight) and then poured into a mixture of ice and 1.0 N HCl (100 mL) and extracted with EtOAc. The extract was washed with 1.0 N HCl (100 mL) and aqueous Na_2CO_3 (200 mL), and the organic solvent dried over anhydrous Na_2SO_4, filtered and evaporated via rotary evaporation. The residue was recrystallized from MeOH to afford yellow crystals (4.7 g, 78%). Crude was further purified through silica gel column chromatography (eluent = DCM) to afford **(11)** as light yellow solid (3.5 g, yield = 70%). ^{1}H NMR (400 MHz, $CDCl_3$): δ 3.84 (s, 3H), 3.89 (s, 3H), 6.70 (s, 1H) 6.93 – 7.01 (m, 3H), 7.07 – 7.11 (m, 1H), 7.21 (d, J = 3.2 Hz 1H,), 7.90 – 7.93 (m, 2H), 11.67 (s, 1H), 15.87 (s, 1H) ppm.

3-fluoro-6-methoxy-2-(4-methoxyphenyl)-4H-chromen-4-one (13): A mixture of **11** (13.6 g, 45.2 mmol, 1.0 equiv) and Selectfluor (**12**, 19.2 g, 54.3 mmol, 1.2 equiv) was stirred with anhydrous CH_3CN (35mL) at RT (15 hr). Concentrated H_2SO_4 (4.50 mL) was then added dropwise to the mixture and the resulting mixture was continuously stirred at RT (0.5 h). The mixture was poured into H_2O and neutralized with NaOH (10%, 90 mL). The solution was extracted with DCM (40 mL × 3). The organic layer was combined and dried over Na_2SO_4. The material was filtered, and the volatiles were removed under reduced pressure and the oil residue was purified by column chromatography (Hex/EtOAc 9:1) to afford **13** as a white solid (10.0 g, 74% yield). ^{1}H NMR (400 MHz, $CDCl_3$): δ 3.90 (s, 3H), 3.92 (s, 3H), 7.04 – 7.08 (m, 2H), 7.28 – 7.32 (m, 1H), 7.49 (d, J = 9.2 Hz 1H,), 7.62 – 7.64 (m, 1H), 7.99 – 8.02 (m, 2H) ppm; ^{19}F NMR (376 MHz): δ -162.8 (s, 1F).

3-fluoro-6-hydroxy-2-(4-hydroxyphenyl)-4H-chromen-4-one (14): Boron tribromide (7.56 mL, 79.92 mmol, 6 equiv) was added to a stirred solution of 3-fluoro-6-methoxy-2-(4-methoxyphenyl)-4H-chromen-4-one (**13**, 4.0 g, 13.32 mmol) in dry CH_2Cl_2 (200 mL) under N_2 at -10 °C. Upon complete addition of BBr_3, the reaction was maintained at -10 °C for 30 min and then allowed to reach RT and stirred (16 h). The mixture was cooled to 0 °C and carefully quenched with H_2O (200 mL). The resulting precipitate was filtered and washed with water (200 mL). Drying under high vacuum gave final compound **14** (3.0 g, 83 % yield). ^{1}H NMR (400 MHz, DMSO d_6): δ 7.00 (d, J = 8.8 Hz, 2H), 7.26 – 7.29 (m, 1H), 7.33 – 7.37 (m, 1H), 7.65 (d, J = 8.8 Hz, 1H), 7.87 (d, J = 8.4 Hz, 2H) 10.15 (s, 1H), 10.40 (s, 1H); ^{19}F NMR (376 MHz): δ -164.7 (s, 1F); ^{13}C NMR (100 MHz): δ 107.66 (d, J = 3.8 Hz), 116.41, 119.47 (d, J = 4.8 Hz), 120.36, 123.89, 124.98 (d, J = 7.3 Hz), 130.39 (d, J = 7.7 Hz), 143.75, 146.15, 148.80, 151.02, 151.26, 155.23, 161.04, 169.55 (d, J = 16.3 Hz). LC/MS-MS (ESI +) 273.1 → 196.9 *m/z*.

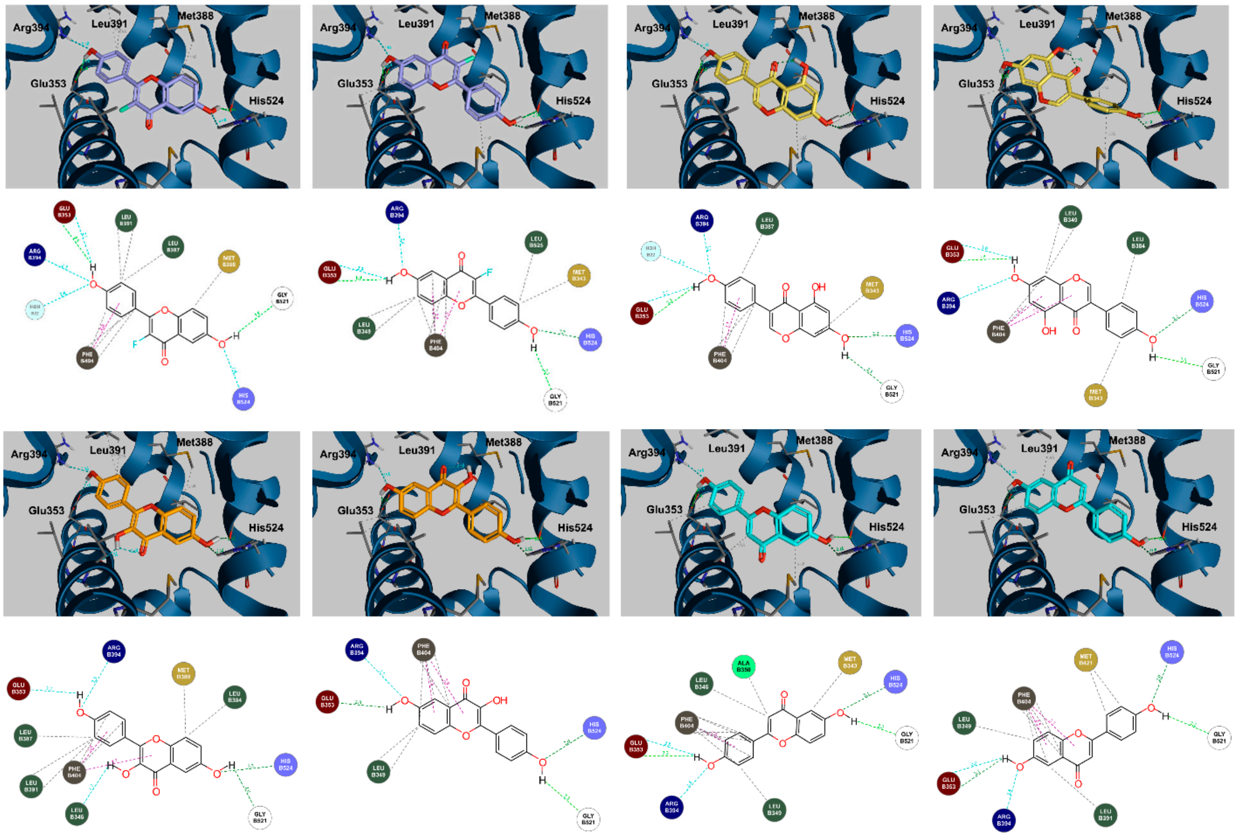

Supplementary Figure S1: Each pair depicts the 'expected' (left) and 'flipped' (right) binding mode of a flavonoid. Upper left, 3-fluoro 6,4′-dihydroxyflavone; upper right, genistein (5,7,4′-trihydroxyisoflavone) lower left, 3,6,4′-triihydroxyflavone, lower right, 6,4′-dihydroxyflavone.
